# Scent of a Dragonfly: Sex Recognition in a Polymorphic Coenagrionid

**DOI:** 10.1371/journal.pone.0136697

**Published:** 2015-08-25

**Authors:** Francesca Frati, Silvana Piersanti, Eric Conti, Manuela Rebora, Gianandrea Salerno

**Affiliations:** 1 Dipartimento di Scienze Agrarie, Alimentari e Ambientali, University of Perugia, Perugia, Italy; 2 Dipartimento di Chimica, Biologia e Biotecnologie, University of Perugia, Perugia, Italy; United States Department of Agriculture, Beltsville Agricultural Research Center, UNITED STATES

## Abstract

In polymorphic damselflies discrimination of females from males is complex owing to the presence of androchrome and gynochrome females. To date there is no evidence that damselflies use sensory modalities other than vision (and tactile stimuli) in mate searching and sex recognition. The results of the present behavioural and electrophysiological investigations on *Ischnura elegans*, a polymorphic damselfly, support our hypothesis that chemical cues could be involved in Odonata sex recognition. The bioassays demonstrate that males in laboratory prefer female to male odour, while no significant difference was present in male behavior between stimuli from males and control. The bioassays suggest also some ability of males to distinguish between the two female morphs using chemical stimuli. The ability of male antennae to perceive odours from females has been confirmed by electrophysiological recordings. These findings are important not only to get insight into the chemical ecology of Odonata, and to shed light into the problem of olfaction in Paleoptera, but could be useful to clarify the controversial aspects of the mating behavior of polymorphic coenagrionids. Behavioural studies in the field are necessary to investigate further these aspects.

## Introduction

The ability of an organism to recognize other individuals in the population can be of biological importance in many social interactions, especially those involving mate choice decisions [1]. Individuals from a wide range of invertebrates and vertebrates are known to discriminate between one’s own and the opposite sex using sex-specific visual, acoustic or chemical cues, and often mate choice is based on multivariate traits perceived in more than one sensory modality [2].

In the Odonata mating system, communication comprises a visual and a tactile stage [3]. The discrimination between potential mates and conspecifics or heterospecifics consists of an initial visual recognition step made by the male as he responds to and approaches a female. This first recognition based on visual stimuli is often inaccurate and males may often try to copulate with heterospecific females [4], who can then reject them [5–7]. Visual cues include body shape [8], coloration pattern [9–12], or displays [5, 7, 10, 13, 14]. The visual recognition is followed by a tactile recognition step involving the male anal appendages in contact with females' pronotum once she is clasped in tandem ("lock and key" hypothesis) [3].

In coenagrionids, discrimination of females is complex owing to the presence of polymorphism where one female morph (androchrome) typically resembles the conspecific male, while the other (gynochrome) does not [15, 16]. For intraspecific recognition of mates, the presence of a learned search image has been proposed [16, 17] to explain why males may preferentially form tandems with the most abundant color morph. On this account, Van Gossum et al. [18] showed that, in artificial conditions, males of *I*. *elegans* can even switch in mate choice between the opposite sex and the same sex by changing the social context. Female polymorphism may have evolved to reduce male harassment (e.g. [19–21]) and males could have faster access to a mate recognizing the more common female morph. As a consequence of this behaviour, the female morph less frequent in the population will receive less male attention [22]. Despite this, the mechanism behind the maintenance of coenagrionids colour polymorphism is still controversial. Indeed in *I*. *elegans*, at variance with Van Gossum's results, Cordero and Sánchez-Guillén [23] found no evidence for males switching their preferences in a frequency-dependent way, suggesting that male attention to female morphs could be highly modified by the experimental setting as previously evidenced by Cordero and Andrés [24].

Despite much research on mating behavior of polymorphic coenagrionids (e.g. [11, 12, 18, 21, 22, 25–31]), it remains understudied which precise cues are relevant to mate-searching males for discriminating among potential mates [32]. In consideration of the remarkable power of vision of Odonata, many studies have focused their attention on visual stimuli (e.g. [10, 33]) and, to date, there is no evidence that damselflies use modes of communication other than vision (followed by the tactile stimuli, as above reported) in mate searching and sex recognition. Moreover a recent experiment with *Enallagma* damselflies suggest that under field conditions, even at close range (i.e. within 10 cm.), olfaction is unlikely to be important in male mate recognition [33].

Although Odonata have been traditionally considered anosmic, ultrastructural investigations revealed the presence of coeloconic sensilla located in pits on the antennal flagellum of different species belonging to Anisoptera and Zygoptera, with a morphology similar to that of olfactory sensilla [34–36]. Electrophysiological studies (Single Cell Recordings-SCR, Electroantennography-EAG) stimulating the antennae of adults of the dragonfly *Libellula depressa* Linnaeus (Odonata: Libellulidae) and of the damselfly *Ischnura elegans* Vander Linden (Odonata: Coenagrionidae) with some generic odors showed that Odonata antennae possess functional olfactory sensory neurons (OSNs) [37, 38]. In addition, a neuroanatomical investigation on the brain of the dragonfly *L*. *depressa* revealed that the antennal sensory neurons project to an aglomerular antennal lobe showing spherical knots that could allow for the perception of odor [39]. A first evidence of the use of olfaction in Odonata behavior has been given by a recent investigation [38] demonstrating by means of behavioral and electrophysiological assays in the laboratory that adults of the damselfly *I*. *elegans* are attracted by olfactory cues emitted by prey that they perceive through olfactory antennal sensilla.

We hypothesize an involvement of olfactory cues in Odonata sex recognition. To verify this hypothesis we used behavioural and electrophysiological (EAG) investigations. The research was conducted in laboratory to simplify and standardize the experimental conditions and to avoid the effect of environmental noise of natural conditions. As in our previous papers [38, 40], our model species is the coenagrionid *I*. *elegans*, a damselfly that can be reared in the laboratory [41]. In this species three female phenotypes occur, one of which has the same blue coloration as the male (androchrome) whilst the others are inconspicuous brown gynochromes (*infuscans* and *infuscans-obsoleta* morphs) [42].

Behavioural laboratory assays are performed to evaluate the following three hypothesis regarding the involvement of chemical volatile cues in:

- the ability of males to recognize the females (males show a preference when tested towards females but without significant difference respect to the control);
- the capacity of males to distinguish androchrome from gynochrome females (males show a different preference for the two tested morphs);
- the possibility of males raised with one female morph or males to exhibit a learned sensory bias for that morph or males (males, raised with one female morph or males, show a preference when tested towards that female morph or males).

EAG recordings on male antennae have been performed in order to evaluate the hypothesis that males OSN are able to respond to olfactory cues from androchrome and gynochrome females and males.

## Materials and Methods

### Insects

*Ischnura elegans* were reared at 25 ± 2°C, a LD 16:8 h photoperiod and 60–80% RH conditions, as described in Piersanti et al. [41]. F0 field founders were males and females collected in late summer along shoreline vegetation in a small artificial pond for fish farming, close to the Trasimeno Lake (Umbria, Central Italy). The pond for fish farming was located in the “Centro Ittiogenico Provinciale del Trasimeno (Perugia, Italy)” and we obtained the permission to collect the insects by the “Provincia di Perugia”. *I*. *elegans* is not a protected species and there are not restriction to collect and to carry out study on this species. The larval stages obtained from eggs laid by F0 founders were reared in aquaria and fed ad libitum with *Artemia salina* nauplii and freshwater plankton (*Daphnia* sp., *Cyclops* sp.). After the emergence males and females were placed together (∼50 individuals) and they were reared in small insectaries (50 x 50 x 50 cm wooden cages covered with bee netting) with 1:1 sex ratio. Adults were fed ad libitum with *D*. *melanogaster* flies. Insectaries and aquaria were provided with artificial solar illumination (36 W/94 Philips TLD, The Netherlands).

In the damselfly *I*. *elegans* the differences between the two gynochromes (*infuscans* and *infuscans-obsoleta*) are minor, and considering that males do not distinguish visually between the two morphs [17], both were pooled in this study and we only considered androchrome and gynochrome females.

All the insects used in the experiments belonged to the F1 generation showing a female frequency of 75% gynochromes and 25% androchromes.

### Male exposure to social contexts

Sexually mature males (about after 6 days from the emergence), maintained with females as described above, were separated and placed in other insectaries (50 x 50 x 50 cm wooden cages covered with bee netting), with a 1:1 sex ratio, to expose them to particular social contexts that could change their sex choice according to Van Gossum et al. [18]. In particular males were maintained in the following four social contexts: a) **males raised with gynochromes + androchromes** together (75% gynochromes and 25% androchromes. Androchrome frequency applied was that observed in our rearing conditions); b) **males raised with only gynochromes**; c) **males raised with only androchromes** and d) **males raised with only males**.

Each time an insect died, it was replaced to maintain constant densities (∼ 50 individuals) and frequencies of female morphs. Insects were used for the bioassays after 4 days of experience in the social context.

### Behavioural assays

To assess whether *I*. *elegans* males responded to volatiles from females and whether they were able to distinguish gynochrome and androchrome female odour, one-way olfactometer bioassays were performed using different olfactory cues. The use of olfactometer allowed to test the pure olfactory cues and exclude the possible effects of other stimuli such as visual or vibrational.

The odour source was always represented by 8 contemporary individuals placed in a bottle with a strip of clean net (5x12cm), and the control was a bottle with only a strip of clean net. Eight females were chosen as odor source since preliminary experiments with 4 females as stimuli source did not elicit any male response (residence time in response to females (197.89 ± 71.81) and to control (107.96 ± 34.53) (mean ± SE) was not significantly different (t = 0.62; df = 42; p = 0.540593)).

To evaluate the hypothesis that males could use olfactory cues to recognize the females, males (raised with gynochromes + androchromes) were tested towards: 1) gynochrome + androchrome (3:1) females, 2) males and 3) control.

To evaluate the hypothesis that males could use olfactory cues to distinguish androchromes from gynochromes and the hypothesis that males could exhibit an olfactory learned sensory bias for one morph or males, males raised in different social contexts were tested. In particular, males raised with gynochromes and those raised with androchromes were assayed towards: 1) gynochrome females, 2) androchrome females and 3) control; males raised with males were tested towards 1) gynochrome females, 2) androchrome females, 3) males and 4) control.

Males and females were isolated individually 1h before the experiments inside two different plastic pots (12 x 12 x 20 cm), filled with moist blotting paper to sustain humidity and they were transferred to the bioassay room. The experiments were performed in the laboratory between 9.00 and 15.00 h under controlled temperature and humidity (25 ± 2°C and 40% RH).

**Fig 1:**
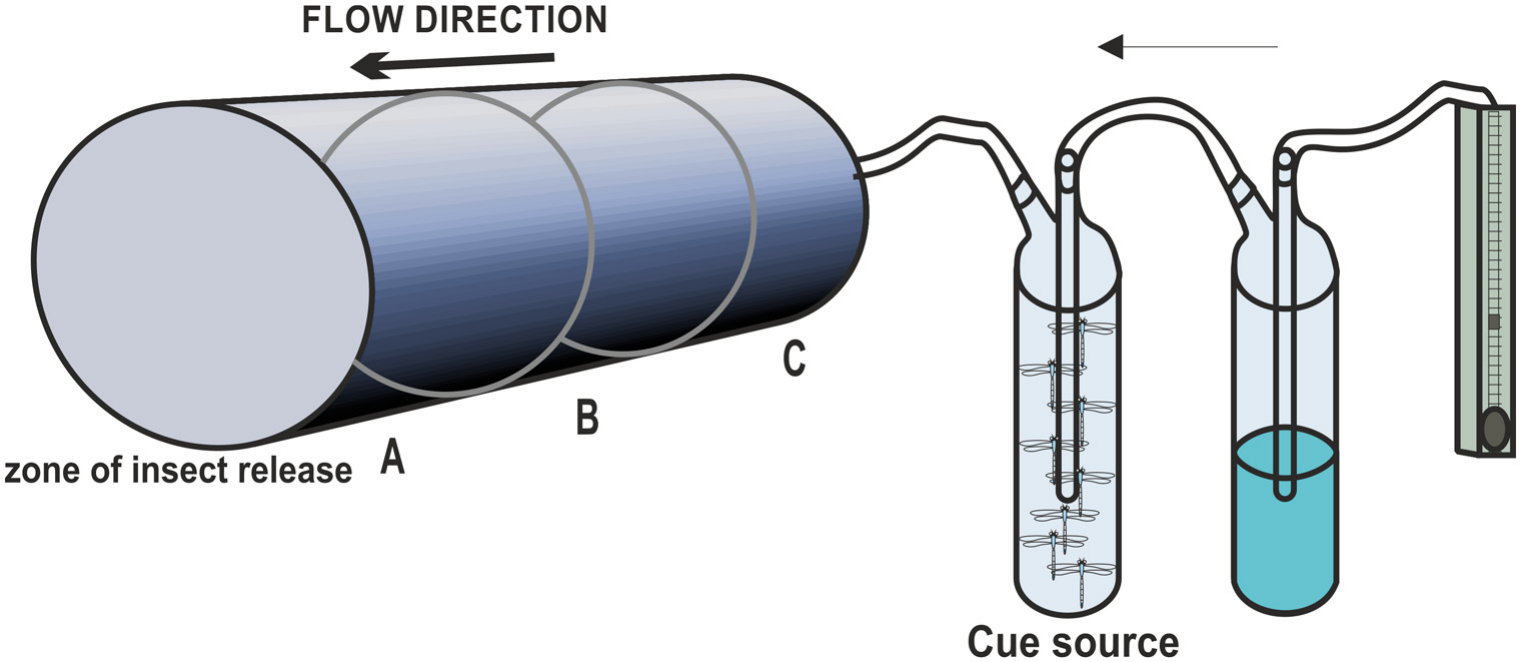
Graphical scheme of the one-way olfactometer. The olfactometer is represented by a glass cylindrical flight chamber into which an individual male is manually released. The air is filtered by a charcoal filter, humidified by bubbling through a glass bottle with distilled water and dispersed by passing through a nylon net. A glass gas washing bottle (300ml) contains the olfactory cues (insects or control). The length of the flight chamber is virtually separated into three equal sectors: A) where the airflow with the olfactory stimuli enters the olfactometer, C) where male is released, and B) positioned between the other two.

The olfactometer was a glass cylindrical flight chamber (diameter = 14 cm, length = 100 cm) into which individual males were manually released 2 cm downwind from the exit of the air (Fig 1). The wind speed checked with both an anemometer (TA 6000; Air Flow Developments Ltd, U.K.) and smoke tubes, was 0.1 m/s. The airflow, checked and regulated using a flow meter, was 6 L/min. Before entering the flight chamber, the air was filtered by means of a charcoal filter, humidified by bubbling through a glass bottle with distilled water and dispersed by passing through a nylon net. The airflow passed through a glass gas washing bottle (300ml) containing the olfactory cues before entering the flight chamber. Under the flight chamber floor, two black strips marked the separation of the chamber into three equal sectors (33 cm long): one where the airflow with the olfactory stimuli entered the olfactometer, the last third where the damselfly was released, and the last was positioned between the other two. The olfactometer was placed inside a metal framework sustaining a black curtain all around to avoid any bias due to differences in the olfactometer sides. A small opening in the front of the curtain allowed to observe the damselfly behavior inside the olfactometer. Six high frequency lamps (TLD 58 W/840 HF, Philips, The Netherlands) were placed above the flight chamber. Each male was observed for 20±1 min and it was used only once. The glass gas washing bottles containing the olfactory cues and those containing the distilled water were placed outside the black curtain. Male behaviour (walking, staying, flying in the three different sectors) was recorded and analysed with an event-recording computer programme (Observer 3.0; Noldus Information Technology, The Netherlands). At the end of each bioassay, the flight chamber was ventilated for 5 minutes. At the end of each series of experiments (8 assays) the odour source was changed and the bottle containing the odour source was washed with water and detergent, wiped with acetone and hexane and baked at 120°C for two hours, whereas the flight chamber was cleaned with acetone.

The analyzed parameters were the residence time (s) in the sector closer to the stimulus and the number of insects reaching the sector closer to the stimulus (%), as a percentage of total tested insects.

### Electroantennography

For EAG recordings mated males (∼10 days old) raised with gynochromes + androchromes (75% gynochrome and 25% androchrome) were employed. The EAG was carried out to evaluate male response towards extracts of gynochromes, androchromes and males. Extracts were obtained by immersing 3 anesthetised adults in a glass vial (4 ml) with 3 ml of hexane (Fluka) for 1h at 24°C. After removal of the insect, the resulting extracts were evaporated under a gentle nitrogen stream to reduce the solvent volume to 750 μl. Then the extracts were kept in a freezer at -18°C until used. A total of 3 extracts for each sex and morph were used.

The *I*. *elegans* male was placed inside a channel (diameter 4 mm) drilled through a Plexiglas cube (side 22 mm) and immobilized by Patafix (UHU Bostik, Milano, Italy) and adhesive tape. The antennal tip of the antenna used for the EAG recordings was cut off. As electrodes two glass capillary (1.5mmo.d, 1.2mmi.d.) filled with Ringer solution [43], containing 5 g/l of polyvinylpyrrolidone (Fluka), in contact with a silver wire, were used. The capillary tubes were drawn to a fine point using the microelectrode puller PC-10 (Narishige, Tokyo, Japan) to get an inner diameter wide enough to enable insertion of antenna. The reference glass electrode was inserted at the pedicellum level and the recording electrode was connected to the antennal tip. The analog signal was detected through a probe with a high-input impedance preamplifier (10x) (EAG Kombi-probe, Syntech, Germany), and was captured and processed with a data acquisition controller (IDAC 4, Syntech, Germany) and analyzed using EAG 2000 software (Syntech, Germany).

Ten microliters of extract were placed on a filter paper (15 mm x 15 mm). The impregnated filter paper was placed into a glass Pasteur pipette (150 mm in length) constituting an odor cartridge. The control stimulus consisted of a similar pipette containing a filter paper impregnated with 10 μl of hexane. Fresh stimulus pipettes were prepared every day. The tip of the glass pipette was placed about 3 mm into a small hole in the wall of a L-shaped glass tube (130 mm long, 12 mm diameter) oriented towards the antennal preparation (∼5 mm away from the preparation). The stimuli were provided as 1 s puffs of purified, charcoal-filtered air into a continuous humidified main air stream (up to 60% RH obtained by bubbling air through a washing glass bottle) at 2500 ml min^-1^, that was flowing over the antennal preparation at a velocity of 50 cm/s generated by an air stimulus controller (CS-55, Syntech, Germany). At least 1 min interval was allowed between successive stimulations for antenna recovery. For each recording series, a pipette with only filter paper was also used to check any contamination of filter paper and to evaluate the air effect in the EAG response. The extracts were presented in a random sequence. Recordings from 16 males were considered.

### Statistical analysis

In the bioassays in one-way olfactometer, to evaluate the hypothesis that males could use olfactory cues to recognize the females, data were analysed using the one-way analysis of variance (ANOVA) with the residence time (s) in the sector closer to the stimulus as dependent variable and the different olfactory cues (gynochrome + androchrome females, males and control) as independent variables. Multiple comparison analyses were limited to two planned orthogonal comparisons that were based on specific a priori predictions.

In the bioassays to evaluate the hypothesis that males could use olfactory cues to distinguish androchromes from gynochromes and the hypothesis that males could exhibit an olfactory learned sensory bias for one morph, data were analysed using the two-way factorial analysis of variance (ANOVA), considering the residence time (s) in the sector closer to the stimulus as dependent variable and the stimuli (female morphs and control) and the social context (males raised with gynochromes, males raised with androchromes, males raised with males) as the main factors. F tests were used to assess the significance of the effects and their interactions. When the effect of social context or the interaction between stimuli and social context were not statistically significant, the data for the different social contexts were merged. Multiple comparison analyses were limited to two planned orthogonal comparisons that were based on specific a priori predictions. Data of EAG investigations were analyzed using the one-way analysis of variance (ANOVA) considering the EAG responses as dependent variable and the different stimuli (extracts of gynochromes, androchromes and males and hexane as control) as independent variables. Multiple comparison analyses were limited to three planned orthogonal comparisons that were based on specific a priori predictions. In the case of the males raised with other males in response to males and in the case of the preliminary bioassays using only 4 females as stimuli source the residence time (s) was analyzed using the *t* test for unpaired comparisons. Before all the analysis described above, Box—Cox transformations were used to reduce data heteroscedasticity [44, 45].

In all one-way olfactometer bioassays, the difference among the number of insects (expressed as percentage in the graphs) reaching the sector closer to the stimulus in the presence of the different stimuli was tested using the X^2^ test and Goodmans’ post hoc procedure for multiple comparisons [46].

## Results

### Behavioural assays

**Fig 2:**
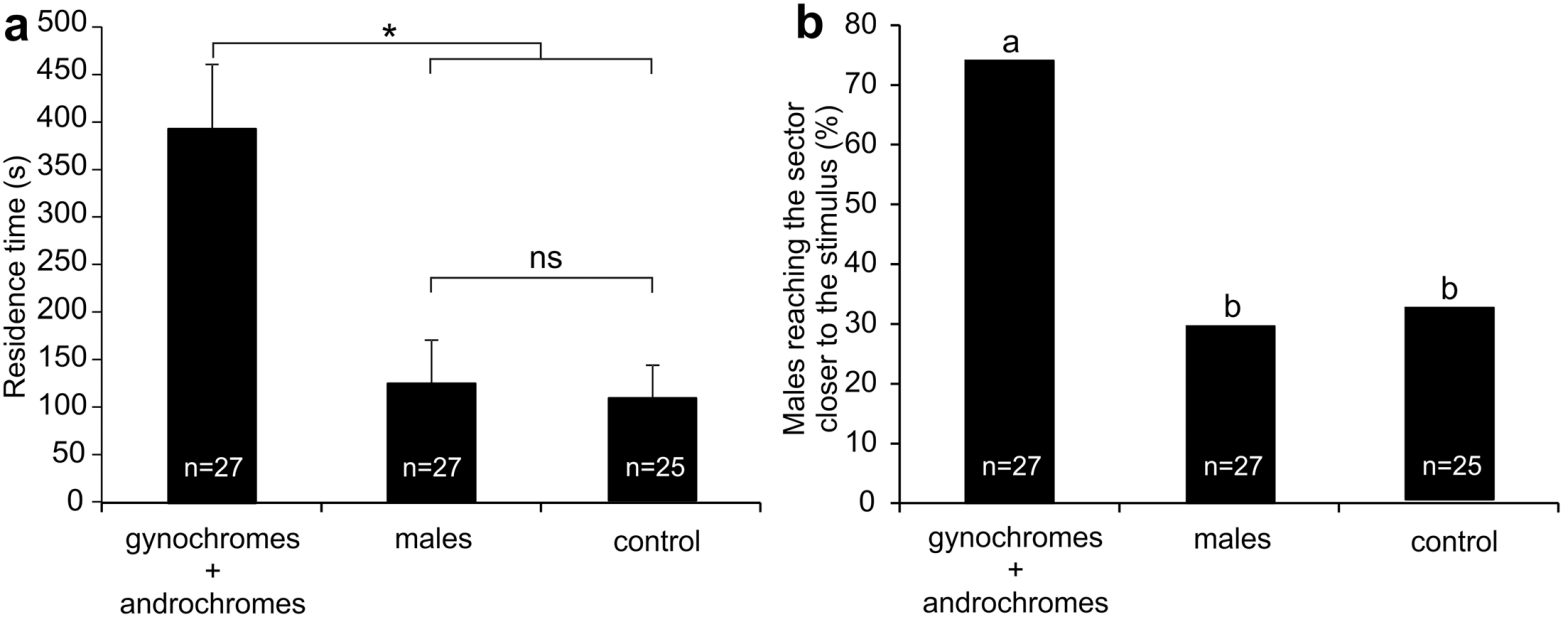
One-way olfactometer response of *I*. *elegans* males raised with gynochromes + androchromes. The males were tested towards: 1) gynochromes + androchromes (3:1), 2) males and 3) control. (a) residence time (s) of males in the sector closer to the stimulus and (b) number of males (% of total tested insects) reaching the sector of stimulus emission. Bars in (a) indicate the means ± s.e.m. n = sample size. ((a): *p < 0.05, ns = not significant; ANOVA, planned orthogonal comparisons for multiple comparison; (b): Data with different letters are significantly different at p < 0.01; X^2^ test, Goodmans’ post hoc procedure).

In the bioassays with the one-way olfactometer, to evaluate the hypothesis that males could use olfactory cues to recognize the females, the males showed significant different responses towards females (gynochromes + androchromes) and males in terms of residence time (s) in the sector closer to the stimulus (F = 661.58; df = 2, 76; p = 0.002) (Fig 2a) and number of males (%) reaching the sector closer to the stimulus (Χ^2^ = 13.47; df = 2; p = 0.0012) (Fig 2b). In particular, the residence time (s) of the males and the percentage of insects reaching the sector closer to the stimulus were significantly higher in the presence of the females than in presence of males or control (Fig 2a and 2b). No significant difference was present in the male behavior between the stimuli from males and the control (Fig 2a and 2b).

**Fig 3:**
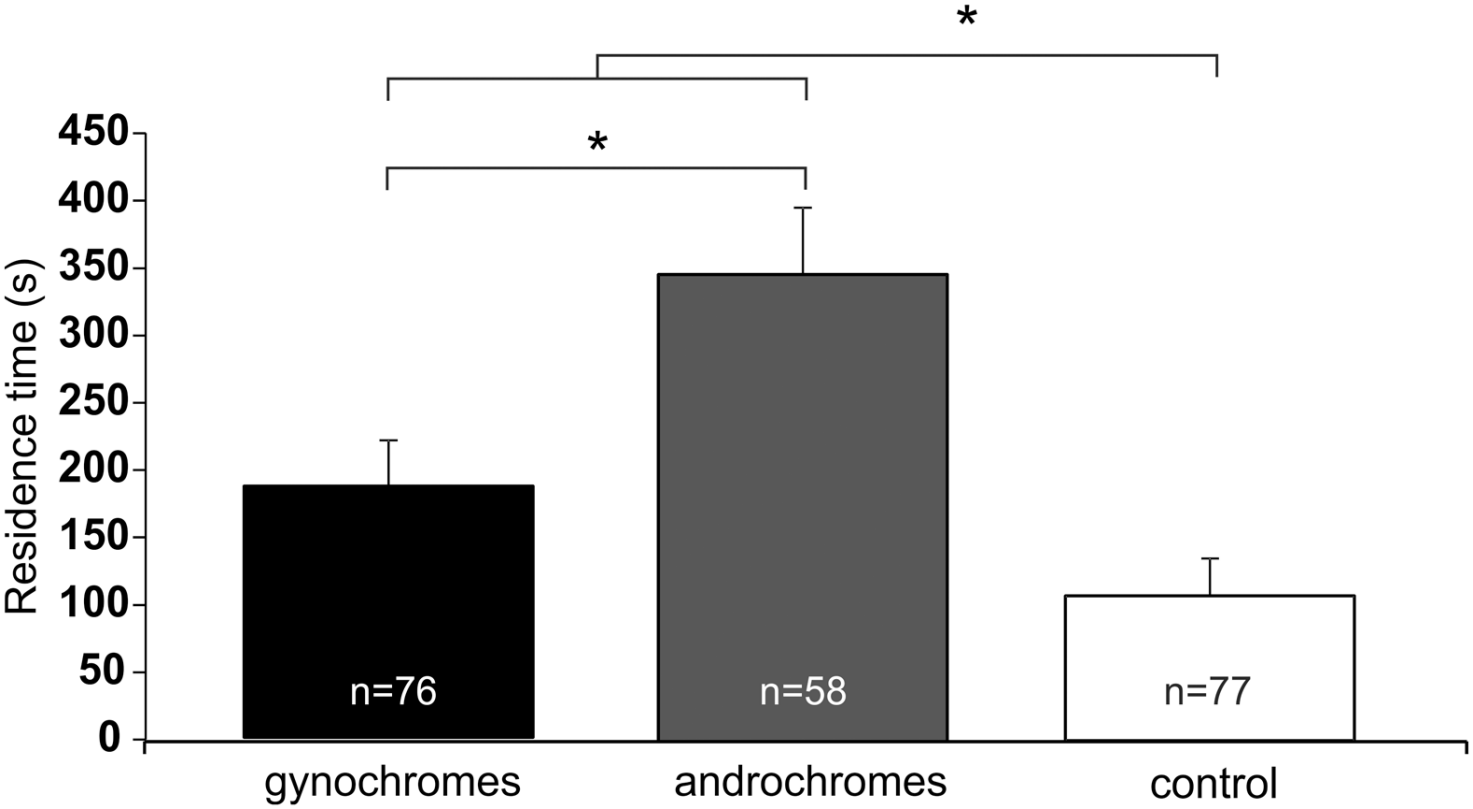
One-way olfactometer response of *I*. *elegans* males raised in different social context. Residence time (s), in reaching the sector closer to the stimulus. Males were tested towards: 1) gynochromes, 2) androchromes, and 3) control. Data for the different social contexts were merged because the effect of social context or the interaction between stimuli and social context were not statistically significant in the two-way ANOVA. Bars indicate the means ± s.e.m. n = sample size. *p < 0.05. (ANOVA, planned orthogonal comparisons for multiple comparison).

The bioassays to evaluate the hypothesis that males could use olfactory cues to distinguish androchromes from gynochromes and the hypothesis that males could exhibit an olfactory learned sensory bias for one morph or males, showed significant differences in terms of residence time (s) in the sector closer to the stimulus among the tested stimuli (gynochromes, androchromes and control) (F = 15.047; df = 2, 202; p < 0.001). No differences were displayed considering the different raising conditions (F = 0.554; df = 2, 202; p = 0.575), and in the interaction between the stimuli and the raising conditions (F = 0.151; df = 4, 202; p = 0.999) (Fig 3). In particular the male response towards gynochromes and androchromes was higher compared to the control and the response towards gynochromes was significant lower compared to androchromes (Fig 3).

**Fig 4:**
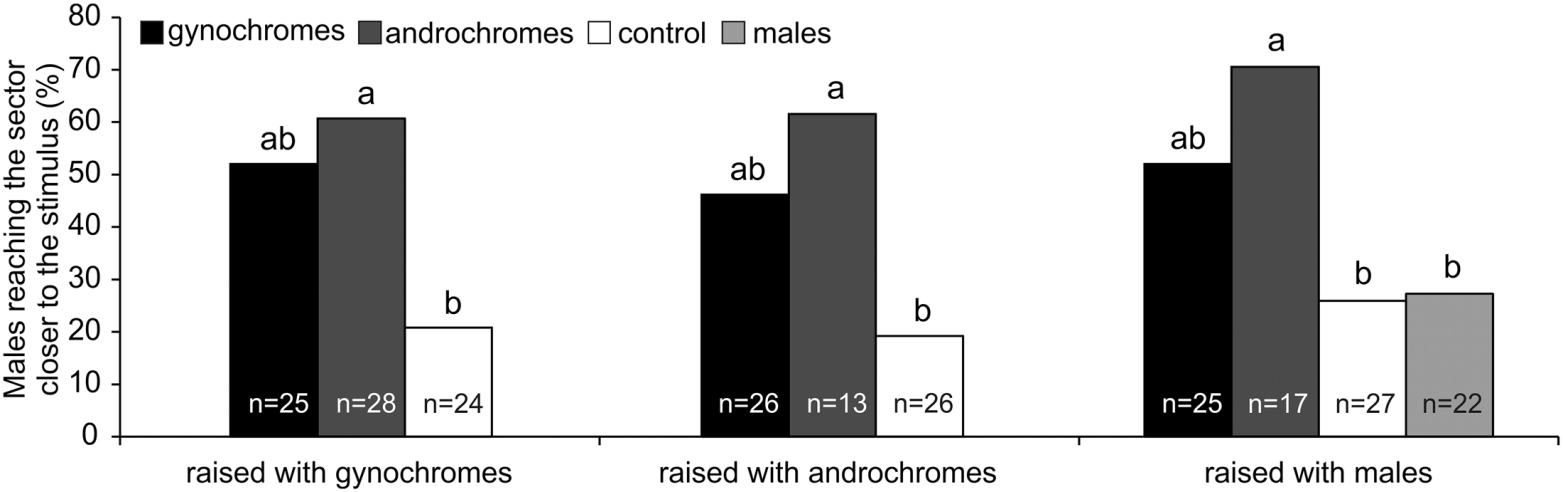
One-way olfactometer response of *I*. *elegans* males raised in different social context. Number of males (% of total tested insects) reaching the sector closer to the stimulus. Males were tested towards: 1) gynochromes, 2) androchromes, 3) males (only for males raised with males), and 4) control. n = sample size. Data with different letters are significantly different at p < 0.05 (X^2^ test, Goodmans’ post hoc procedure)

Moreover, the residence time (s) of the males raised with other males in response to males (81.55 ± 32.48) and control (79.52 ± 34.57) (mean ± SE) was not significantly different (t = -0.143; df = 47; p = 0.887). In males raised either with gynochromes (Χ^2^ = 8.93; df = 2; p = 0.0115) or androchromes (Χ^2^ = 7.64; df = 2; p = 0.0230), the number of males (%) reaching the sector closer to the stimulus was significantly higher when the stimulus was represented by androchromes in comparison with the control, while it was intermediate when the stimulus was represented by gynochromes (Fig 4). In males raised with males the number of insects (%) reaching the sector closer to the stimulus was significantly higher when the stimulus was represented by androchromes in comparison with the males and the control, while it was intermediate when the stimulus was represented by gynochromes. In addition, no significant difference was shown between the responses towards the males and the control (Χ^2^ = 11.57; df = 3; p = 0.0090) (Fig 4).

### Electroantennography

**Fig 5:**
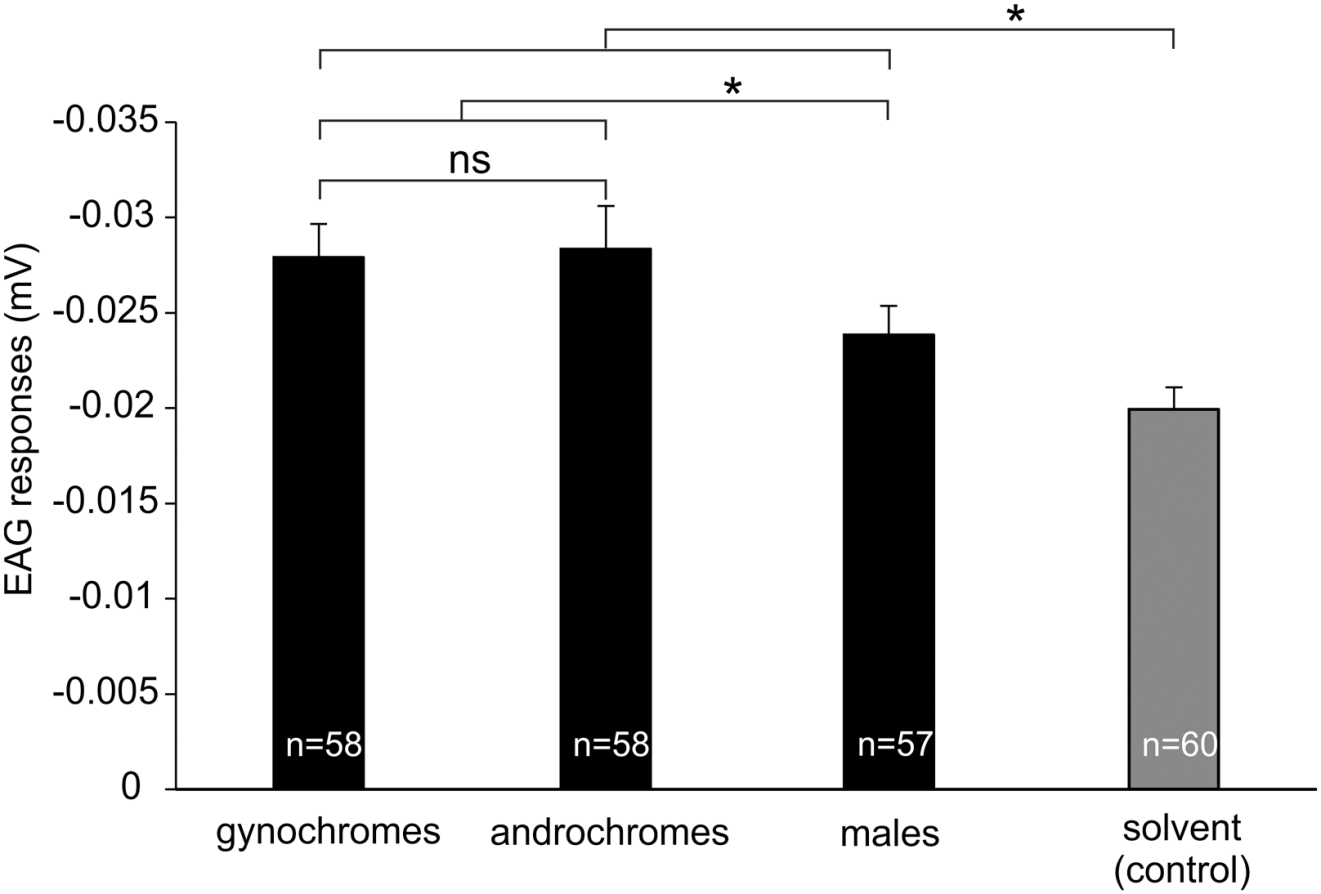
EAG responses of *I*. *elegans* males. Males raised with females (gynochromes + androchromes) were tested towards hexane extracts from gynochromes, androchromes, and males and towards hexane used as control. Bars indicate the means ± s.e.m. n = sample size. *p < 0.05, ns = not significant (ANOVA, planned orthogonal comparisons for multiple comparison).

EAG responses were recorded in males of *I*. *elegans*. In particular, the extracts obtained from gynochrome, androchrome and from males elicited EAG activity significantly higher in comparison with the EAG activity recorded in response to the solvent. The EAG responses elicited by the extracts obtained from gynochrome and androchrome were also significantly higher compared to those elicited by male extract but no difference was recorded between the responses elicited by the extracts of the two female morphs (F = 5.44; df = 3, 229; p = 0.0013) (Fig 5).

## Discussion

The results shown in the present study support the hypothesis that chemical cues could be involved in Odonata sex recognition. Indeed the bioassays demonstrate that, in laboratory conditions, males of *I*. *elegans* prefer female to male odour in terms of residence time in the sector closer to the stimulus and percentage of males reaching the same sector. In addition, no significant difference was present in the male behavior between the stimuli from males and the control.

The fact that potential mates could be detected by males using chemical cues together with visual cues offers new perspectives in Odonata mating studies. Although thus far no sexual pheromone has been documented in any odonate species [47], the female odour acting as a cue in the damselfly *I*. *elegans* could be produced by a gland or, more probably, to be a cuticular hydrocarbon. In insects, chemical communication is often facilitated by cuticular hydrocarbons [48]. Cuticular hydrocarbons (or CHCs) are lipid compounds located on the outermost layer of the epicuticle, useful to prevent desiccation and as a barrier to microorganisms. Besides providing these basic physiological functions, CHCs often play an integral role in insect chemical communication and have been demonstrated to function as important recognition cues facilitating species recognition, kin recognition and sex recognition in a variety of insect taxa [48]. The chemical composition of CHCs consists of long carbon chains, making them ideal recognition cues with low volatility, high chemical stability, and a diversity of structures allowing for significant variation in lipid composition [48, 49]. In fact, in our preliminary experiments (see material and methods section), a low number of females did not elicit any difference in the males behavior, thus suggesting that the female odour could be characterized by a low volatility and that it could be active at a short range. Further investigation aiming to identify the chemical profiles of the different female morphs and of the males are necessary to clarify the characteristics of the chemicals potentially involved in Odonata mate discrimination and to verify if the male ability to distinguish between males and females is due to qualitative or quantitative differences in the chemicals, considering also the differences in size between male and females [50].

The ability of males to perceive odours from the females has been confirmed by our electrophysiological investigation. The extracts obtained from androchromes and gynochromes and males elicited EAG activity significantly higher in comparison with that recorded in response to the solvent, and the extract obtained from the two female morphs elicited an EAG activity significantly different from that obtained from males. In particular some of the antennal OSNs in the coeloconic sensilla of *I*. *elegans* [38] could be involved in female odour perception. The differences in the EAG response of the male of *I*. *elegans* to males and females extracts could be evidenced although the magnitude of the EAG response is fairly weak in this species, such as that obtained in *L*. *depressa* [37, 40], if compared with that shown by other insects. This observation is consistent with the low number of olfactory sensilla located on Odonata antennae, especially in Zygoptera [36]. Weak absolute EAG responses have been recorded in other insects such as *Scaphoideus titanus* (Cicadellidae), characterized by a low number of olfactory sensilla and showing an absolute EAG response to odours from the host plant of about 0.09 mV [51].

In our behavioural investigations with the one-way olfactometer, interestingly, the male response to androchromes in terms of residence time in the sector closer to the stimulus was higher than to gynochromes. Similarly, when considering the number of males reaching the sector closer to the stimulus, it was significantly higher when the stimulus was represented by androchromes in comparison with the control, while it was intermediate when the stimulus was represented by gynochromes. These data make us to hypothesize some ability of the males to distinguish between the two female morphs on the basis of chemical stimuli. This hypothesis has been considered in a study on the polymorphic damselfly *Nehalennia irene* Hagen; in this study male mate preference to natural and manipulated female morphs was scored and, in contrast to expectation, male preference did not change when colour was manipulated, thus suggesting that cues other than body coloration primarily affect male mate preference [32]. In another study in the field on *I*. *elegans* the authors hypothesizes that males could use many other cues rather than colour to distinguish between the two female morphs, indeed in this study there is clear evidence that males behave indiscriminately to female morphs when the stimuli are dead females instead of alive ones [42].

On the basis of our results, where the residence time and the number of insects reaching the sector closer to the stimulus were significantly higher when the stimulus was represented by androchromes in comparison with the males, we have to rule out a possible chemical mimicry between androchromes and males hypothesized by Cordero et al. [42]. Our results are in agreement with the observations of Fincke et al. [52] on the coenagrionid *Enallagma civile*, where even males reared only with males rarely reacted sexually toward other males, and on *Enallagma ebrium* under field conditions, where males that experience females took androchromes in tandem more often than males, in contrast with studies indicating that males do not distinguish between androchromes and males [19, 53]. The ability to distinguish between androchromes and males is apparently in contrast with Van Gossum et al. [18] observations where, after a 2-day exposure to only males, males of *I*. *elegans* preferentially formed tandems with other males. Anyway, we have to consider the extreme social context of these experiments, not existing in natural populations and therefore very different from behaviour in field conditions as already suggested for similar studies by Cordero and Andrés [24]. Moreover, in our bioassays to evaluate the possibility of males raised with one female morph (androchromes or gynochromes) or males to exhibit a learned sensory bias for that morph or males we observed no differences in the males behavior considering the different raising conditions, thus suggesting that the learned sensory bias for one morph evidenced in polymorphic damselflies [16–18] is not based only on olfactory stimuli.

Odonata have highly acute vision [54, 55] and their ability to detect UV and a range of colors [56, 57] make them visually oriented insects but other kind of stimuli, especially at short range should not be dismissed out of hand.

In polymorphic damselflies, discriminating between the sexes when one sex resembles the members of the other sex may be challenging. When sexual mimicry imposes costs on signal receivers, receivers can minimize confusion by using nonmimetic cues that differ between the models and the mimics. Xu et al. [12] testing this hypothesis in *Enallagma hageni*, showed the use of colour (the mimetic cue) and pattern (the nonmimetic cue) in sex recognition: males use the nonmimetic cue only when the encountered individual has the mimetic colour. Our experiments suggest that in *I*. *elegans* the odour (probably acting at close range) could represent a nonmimetic cue particularly useful for males to distinguish androchrome females. Similarly, in a study on the lizard *Platysaurus broadleyi*, female-mimicking males (“she-males”) were able to mimic visual, but not chemical, signals of females; consequently, “he-males” court “she-males” using at long distance only visual signals, while, at closer range chemical signals become detectable and it becomes less probable that “he-males” court she-males [58].

The sense of smell of Odonata is surely poor in comparison with that of many other insects, also in consideration of the simple organization of the antennal lobe [39]. However, its involvement in the perception of the prey has been shown in behavioural and electrophysiological investigation under controlled conditions [40] and the present study shows in the laboratory the involvement of chemical cues in the ability of males to move towards females (with an increased chemical attractiveness for one morph), recognizing them without any involvement of sight. Our data do not show that odonates in the wild can actually differentiate sex using olfactory cues. In this regard considering the complexity of natural environment, behavioral investigation in the field are necessary and will be performed in the future. These studies are important not only to get insight into the chemical ecology of Odonata, so far disregarded, and to shed light into the problem of olfaction in Paleoptera to trace evolutionary trends in insect chemical communication, but could be useful also to clarify the controversial aspects of the mating behavior of polymorphic coenagrionids. These aspects deserve further attention.

## Supporting Information

S1 Dataset Behavioural and EAG data underlying the findings described in the manuscript. (XLSX)
